# The effects of Anisodamine-Tirofiban Combined Therapy in acute myocardial infarction treated with Percutaneous Coronary Intervention (PCI)

**DOI:** 10.12669/pjms.38.7.5410

**Published:** 2022

**Authors:** Yan Jiao, Xiaoying Fu, Qingxin Liu, Wei Zhang

**Affiliations:** 1 Yan Jiao, Department of Pharmaceutical Analysis, Cangzhou Medical College, Yingbin South Avenue, Yunhe District, Cangzhou 061000, Hebei, China; 2 Xiaoying Fu, Department of Pharmaceutical Preparations, Cangzhou Medical College, Yingbin South Avenue, Yunhe District, Cangzhou 061000, Hebei, China; 3 Qingxin Liu, Department of Pharmaceutical Analysis, Cangzhou Medical College, Yingbin South Avenue, Yunhe District, Cangzhou 061000, Hebei, China; 4 Wei Zhang, Department of Cardiovascular Medicine, Cangzhou Central Hospital, Cangzhou 061000, Hebei, China

**Keywords:** Anisodamine, Tirofiban, Cardiac function, Endothelial cell-specific molecule 1 (ESM-1), Nerve Growth Factor (NGF)

## Abstract

**Objectives:**

To study the effects of anisodamine-tirofiban combined therapy on cardiac function and serological expression of serum NGF and ESM-1 in patients with acute myocardial infarction treated with percutaneous coronary intervention (PCI).

**Methods:**

Eighty patients with myocardial infarction treated in Cangzhou Medical College, Hebei, China from February 2015 to April 2017 were selected and divided into the control group and the research group according to the principle of random draw, 40 patients per group. The patients in the control group received symptomatic routine treatment, while the patients in the research group received anisodamine-tirofiban combined therapy on the top of symptomatic routine treatment. Differences between the two groups in TIMI flow grades, cardiac function, levels of NGF and ESM-1 and adverse response were observed.

**Results:**

The recovery of cardiac function in the research group was statistically significant with P value (p<0.05) and better than the control group in TIMI flow grades, myocardial perfusion capacity and cardiac function. The serological indicators in the research group had a higher level of NGF and a lower level of ESM-1 than the control group, and the differences were statistically significant (p<0.05). In terms of safety, neither group showed significant hepatorenal disorders.

**Conclusion:**

The combined treatment of anisodamine-tirofiban in patients with acute myocardial infarction after percutaneous coronary intervention (PCI) can recover NGF and ESM-1 related proteins, improve postoperative myocardial perfusion, and accelerate the recovery of cardiac function. It is worth promoting in clinic.

## INTRODUCTION

Acute myocardial infarction is one of the common diseases of cardiology. In recent years, the incidence of acute myocardial infarction in China has been on the rise, with new cases of more than 500,000 every year.^1^,^2^ The major triggers include sudden climate change, emotional agitation, overeating, constipation, smoking and alcohol drinking.^3^ At present, percutaneous coronary intervention (PCI) is a primary treatment for acute myocardial infarction, and a large number of studies of evidence-based medicine have proved that PCI can effectively reduce the mortality rate of patients with acute myocardial infarction. However, the recipients generally show poor myocardial reperfusion capacity and poor prognosis, and they are prone to arrhythmias, severe heart failure, hypotension, and even life threat.^4^ It may be that the combination therapy of anisodamine-tirofiban after PCI can effectively solve this problem. Anisodamine, a drug with multiple pharmacological effects, can maintain the stability of the hemodynamic states and increase heart rate (HR), blood pressure (BP), coronary perfusion pressure and heart inotropy, making it suitable for acute inferior myocardial infarction (AIMI).^5^ Tirofiban, as a reversible Glycoprotein IIb/IIIa receptor inhibitors (GPIs), blocks the binding of fibrinogen to receptors by selectively blocking GP receptors on the surface of platelets and inhibits platelet activity. Thus preventing stent thrombosis and vascular stenosis after PCI.^6^ Nerve growth factor (NGF) promotes neovascularization whereas endothelial cell-specific molecule 1 (ESM-1) is a marker of inflammatory response in vascular endothelial cells. The above two markers are significant for predicting whether acute myocardial infarction will develop into permanent myocardial infarction.^7^ Although the combination therapy of anisodamine and tirofiban has been applied clinically but documented literature is scarce. This study explores the effects of anisodamine-tirofiban combined therapy on cardiac function and the expression of serum NGF and ESM-1 in patients with acute myocardial infarction who underwent percutaneous intervention (PCI), to provide a scientific basis for clinical treatment.

## METHODS

### General Data

The subjects were 80 patients with ischemic cardiomyopathy heart failure treated in Cangzhou Medical College, Hebei, China from February 2015 to April 2017. Male: 41, female: 39. Age: 55-72. Average age: 66.72±2.37. Gap between attack and hospital admission: thirty minutes to six hour, average gap: (3.12±0.34)hour. The above patients were randomly divided into the control group and the research group according to the principle of random draw, with 40 patients per group. The control group: male: 21, female: 19; age: 55-70, average age: 65.99±2.65; gap between attack and hospital admission: 30min-6h, average gap: (3.17±0.27) hour. The research group: male: 20, female: 20; age: 56-72, average age: 66.78±2.14; gap between attack and hospital admission: thirty minutes to six hour,, average gap: (3.09±0.33) hour. The differences between the two groups in gender, age and gap between attack and hospital admission were not statistically significant (P<0.05), so they were comparable. All patients signed an informed consent and were approved by the hospital ethics committee.

### Ethical Approval

Randomized controlled trial was used in this study. The study was approved by the Institutional Ethics Committee of Cangzhou Medical College on June 2021 (No. [2021]023), Yingbin South Avenue, and written informed consent was obtained from all participants.

### Inclusion Criteria

A. The patient met the pre-hospital diagnostic criteria for acute myocardial infarction^8^; B. was confirmed as acute myocardial infarction by imageological examination such as cardiac magnetic resonance; C. was admitted within 6~72hour after attack; D. had a National Institute of Health stroke scale (NIHSS) score of 5~25; E. was conscious and had no communication disorder; F. an informed consent was obtained from the patient and his/her family members.

### Exclusion Criteria

A. Severe heart disease and hepatorenal dysfunction; B. concurrent malignant tumor; C. hematological system diseases; D. mental disorder, disturbance of consciousness; E. allergic to the study drugs.

### Research Methods

The control group received conventional PCI; the research group received anisodamine-tirofiban combined therapy on the basis of symptomatic routine treatment.

### Conventional PCI

Both groups received 300mg of Plavix (Sanofi Winthrop Industries, SFDA approval number: J20080090) and 300mg of Aspirin Enteric-coated Tablets (Bayer China, SFDA approval number: J20080078) before surgery. 30min later, the surgery was started. During the surgery, guided by a 6F catheter, the coronary artery infarction was located before balloon treatment was performed. Patients that still suffer blood disorders after the coronary artery lumen is opened received 200μg of nitroglycerin pellets. After that, angiography was performed to the coronary artery in 1-5minutes after operation to observe the changes of blood flow.

During the above PCI surgery, each patient of the research group received loading dose of 5μg/kg of tirofiban (LUNAN BEITE Pharmaceutical Co., Ltd., SFDA approval number: H20090225) through intraluminal injection into the coronary artery within four minutes followed by. Maintenance dose of tirofiban 0.075μg/Kg/min for eight hour. At an interval of five minutes after intraluminal injection of tirofiban into the coronary artery, each patient receives 1.5mg of anisodamine (SINOPHARM RONSYN, SFDA approval number: H41023400), injected within two minutes.

All patients received conventional angiotensin converting enzyme inhibitors (ACEI) or other statin lipid-lowering drugs after the surgery.

### Observational Indexes

TIMI flow grades:^9^ Postoperative contrast examinations were carried out to the coronary artery of the patients. If the contrast agent successfully went through the distal vascular bed at the occlusion of the coronary artery, or the blood flow velocities at the coronary artery lesion and in the forward blood vessel were consistent, it was judged as “total perfusion, grade 3”; if the contrast agent successfully went through the lesion of coronary artery and the distal vascular bed of the lesion was completely exposed, and the proximal blood vessel showed a coronary artery bed, it was judged as “partial perfusion, grade 2”; if the contrast agent completely went through the lesion of the coronary artery, but no imaging is found in the distal or proximal end of the occlusion, the result was judged as “through, but no perfusion, grade 1”; if the contrast agent does not went through the occlusion, it was judged as “no perfusion, grade 0”.

### Post-Treatment Cardiac Function

Differences between the two groups in left ventricular ejection fraction (LVEF), left ventricular end-systolic dimension (LVESD), and left ventricular end-diastolic dimension (LVEDD) were observed.

### Comparison of NGF and ESM-1 expression levels before and after treatment

Fasting venous blood (4ml) is sampled from both groups. The sample was centrifuged (3000r, 15min). Then, the ELISA method was used to determine the levels of NGF and ESM-1 in serum. The differences between the two groups were analyzed. The test kits were all provided by Shanghai mlbio Biotechnology Co., Ltd. The differences between the two groups in adverse reaction were also analyzed.

### Statistical Analysis

All data was summarized and analyzed using SPSS 20.0 software. Rank sum test was adopted for comparing TIMI flow grades; chi-square test was adopted for comparing adverse reaction; independent-samples T test was adopted for comparing post-treatment cardiac function and levels of NGF and ESM-1. The measurement data is expressed in (*x̅*±*s*) and the enumeration data is expressed in [n(%)]. P<0.05 means the difference is statistically significant.

## RESULTS

In one to five minutes after operation, significant differences were observed between the two groups in TIMI flow grades (p<0.05), Table-I The two groups were compared in terms of LVEF, LVESD and LVEDD before treatment. It was found that the differences were not statistically significant (P>0.05). So, the two groups were comparable. One month after treatment, the LVEF, LVESD and LVEDD data was significantly improved in both groups, and the differences were statistically significant (p<0.05)). The research group had significantly better cardiac function than the control group. Table-II.

**Table I:** TIMI blood flow classification in two groups of patients [n(%)].

Group	Grade 0	Grade 1	Grade 2	Grade 3
Control group (n=40)	4(10.00%)	7(17.50%)	8(20.00%)	21(52.50%)
Research group (n=40)	1(2.50%)	4(10.00%)	5(12.50%)	30(75.00%)
U value		2.15		
P value		0.03		

**Table II:** Comparison of cardiac function between the two groups (*x̅*±s).

Group	LVEF (%)	LVESD (mm)	LVEDD (mm)
	Before treatment	After treatment	Before treatment	After treatment	Before treatment	After treatment
Control group (n=40)	39.26±4.19	45.27±4.62*	59.81±4.23	54.11±4.28*	58.68±4.11	55.26±4.22*
Research group (n=40)	38.89±4.24	56.73±4.26^#^	60.01±4.11	45.19±4.15^#^	58.25±4.26	50.27±4.95^#^
t value	0.39	11.53	0.21	9.46	0.46	4.85
P value	0.34	0.00	0.42	0.00	0.32	0.00

Note: Compared to before treatment,

* means P<0.05,

# means P<0.01.

Before treatment, the differences between the two groups in the levels of NGF and ESM-1 were not statistically significant (p> 0.05). After treatment, both groups showed a rise of NGF levels and a decrease of ESM-1 levels, which were statistically significant (p<0.05). The research group showed a higher level of NGF and a lower level of ESM-1 than the control group, and the differences were statistically significant (p<0.05). Table-III. After treatment, neither group showed hepatorenal dysfunction.

**Table III:** Comparison of the levels of NGF, ESM-1 in the two groups of patients.

Group	ESM-1(ng/L)		NGF(ng/L)	

	Before treatment	After treatment	Before treatment	After treatment
Control group(n=40)	1.21±0.44	1.16±0.12*	0.21±0.04	0.26±0.02*
Research group(n=40)	1.22±0.34	1.12±0.14^#^	0.22±0.06	0.34±0.06^#^
t value	0.11	2.06	0.34	3.77
p value	0.91	0.04	0.73	0.00

Note: Compared to before treatment,

* means P<0.05,

# means P<0.01.

## DISCUSSION

With the continuous development of society and economy, China has become an aging society. Due to people’s unhealthy lifestyle and aging, the incidence of acute myocardial infarction is increasing day by day.^10^ At present, it is believed that the main cause of acute myocardial infarction is coronary thrombosis, which further causes poor myocardial blood perfusion and then triggers a series of clinical symptoms.^11^ What triggers intraluminal thrombus? At present, a widely recognized answer is endothelial dysfunction. Endothelial dysfunction causes abnormal metabolism of lipids, and promotes the oxidation of lipid proteins and inflammatory response, thus resulting in abnormal smooth muscle proliferation in patients. As endothelial cells fall off and the intraluminal blood lipid concentration continues to rise, the patients are prone to atherosclerosis, which may lead to artery stenosis or blockage, thus causing cardiomyocyte ischemia and a series of clinical symptoms.^12^ Platelet aggregation plays a key role in the process of thrombus formation.^13^ Activated platelets not only participate in collective hemostatic and clotting mechanisms, but also activate a variety of cytokines and nerve growth factors, such as ESM-1 and NGF. ESM-1 is a marker protein of the cellular inflammatory response. It is a good indicator for platelet aggregation and the exfoliation of endothelial cells. And its content is positively correlated with the platelet content of the patient.^14^ NGF is a good indicator for neovascularization in patients. In patients with myocardial infarction, due to serious myocardial ischemia, the cardiomyocytes are unable to get nutrition supply, so some cardiomyocytes suffer necrosis. Whether cardiomyocyte necrosis is permanent or not is of great significance for the prognosis of a patient.^15^ At this time, capillary growth has a positive effect on the reactivation and nutrition supply of cardiomyocytes, and NGF mentioned in this study is an important biological indicator for capillary neogenesis.^16^ Tirofiban has an obvious inhibitory effect on platelet agglomeration, so it has positive efficacy in preventing cardiac ischemia and complications related to sudden coronary occlusion. Anisodamine has obvious peripheral cholinolytic capacity, so it has preventive and therapeutic effects on postoperative hypotension.

In this study, the research group had a significantly higher myocardial complete perfusion rate than the control group after treatment. To be specific, although the control group received PCI and contrast agents went through the coronary artery lesions in some patients, myocardial tissues could not achieve effective perfusion, and the probabilities of arrhythmia, myocardial ischemia and hypotension were high, with poor prognosis.^17^ However, by intraluminal injection of tirofiban, platelet aggregation in the coronary artery could be immediately inhibited, and the smoothness of the coronary artery was guaranteed. Previous studies have also found that tirofiban intravascular injection can effectively improve myocardial reperfusion after emergency PCI in elderly STEMI patients,^18^ which was similar to the results of our study. At the same time, anisodamine could effectively prevent hypotension caused by sudden blood supply to myocardial tissues. Previous studies have shown that anisodamine combined with nicordil can effectively improve myocardial reperfusion and protect cardiac function after PCI for AIMI.^5^ Therefore, overall, the research group had better myocardial perfusion than the control group. In a practice of treating acute myocardial infarction patients with thrombus aspiration in combination with tirofiban by Krishna K et al.^19^, the patient showed significantly improved perfusion capacity of the myocardial tissues and good prognosis, which is similar to the results of this study.

The research group showed significantly better cardiac functions than the control group. After intraluminal administration of the above two drugs, the research group showed improved blood supply to myocardium, steadily improved perfusion capacity, effectively eased the smooth muscle spasm of the coronary artery, effectively inhibited proliferation of smooth muscle cells, further improved myocardial contractility, enhanced systole and diastole, and improved the ejection ability.^20^

The research group had a higher level of NGF, but a lower level of ESM-1 than the control group. To be specific, through intraluminal injection of tirofiban and anisodamine after PCI, the cardiac perfusion capacity was improved, the damage of endothelial cells was gradually repaired, the ability to stimulate the coronary artery decreased, coronary artery spasm was effectively controlled, capillary growth was effectively guaranteed, and the probability of permanent myocardial infarction was significantly reduced, with good prognosis. A study of Wei P^21^ et al on patients with ST segment elevation myocardial infarction showed that the level of serum ESM-1 was significantly reduced, and the endothelial injury was gradually recovered by the use of ticaglelor, which confirms each other with the results of this study.

### Limitations of this study

Since the study was a small randomized controlled trial, these results are not conclusive. Further large-scale trials are needed to confirm these results. The quantitative index correlation between reperfusion amount and drug dosage in MI patients remains to be studied in the future.

## CONCLUSION

To sum up, the combined use of tirofiban and anisodamine in patients with myocardial infarction after operation leads to strong myocardial perfusion capacity and good cardiac function recovery. Meanwhile, it reduces the inflammatory response of blood vessel endothelium by recovering NGF and reducing the ESM-1 related proteins. The patients have good prognosis. The method is worth replicating clinically.

### Authors’ Contributions:

**YJ & XF:** Designed this study, prepared this manuscript, are responsible and accountable for the accuracy and integrity of the work.

**QL:** Collected and analyzed clinical data.

**WZ:** Data analysis, significantly revised this manuscript.
